# Paleoproteomics of the Dental Pulp: The plague paradigm

**DOI:** 10.1371/journal.pone.0180552

**Published:** 2017-07-26

**Authors:** Rémi Barbieri, Rania Mekni, Anthony Levasseur, Eric Chabrière, Michel Signoli, Stéfan Tzortzis, Gérard Aboudharam, Michel Drancourt

**Affiliations:** 1 Aix-Marseille Université, URMITE, CNRS, Faculté de Médecine IHU Méditerranée-Infection, Marseille, France; 2 Aix-Marseille Université, EFS-CNRS, Marseille, France; University of Florence, ITALY

## Abstract

Chemical decomposition and fragmentation may limit the detection of ancient host and microbial DNA while some proteins can be detected for extended periods of time. We applied paleoproteomics on 300-year-old dental pulp specimens recovered from 16 individuals in two archeological funeral sites in France, comprising one documented plague site and one documented plague-negative site. The dental pulp paleoproteome of the 16 teeth comprised 439 peptides representative of 30 proteins of human origin and 211 peptides representative of 27 proteins of non-human origin. Human proteins consisted of conjunctive tissue and blood proteins including IgA immunoglobulins. Four peptides were indicative of three presumable *Yersinia pestis* proteins detected in 3/8 dental pulp specimens from the plague-positive site but not in the eight dental pulp specimens collected in the plague-negative site. Paleoproteomics applied to the dental pulp is a new and innovative approach to screen ancient individuals for the detection of blood-borne pathogens and host inflammatory response.

## Introduction

The discovery and the characterization of microbes in ancient environmental and human specimens expanded the knowledge about the evolution of microbiota and pathogens and rose new paradigms concerning the dynamics of deadly epidemics [1]. New insights into bacteria and archaea constituting past microbiota have been gained notably by analyzing ancient dental calculus microbiota [2–4] and digestive tract microbiota [5]. Furthermore, an expanding knowledge of the evolution of pathogens such as *Yersinia pestis* [6, 7], *Mycobacterium tuberculosis* and *Mycobacterium leprae* [8] and variola [9] helped reconstitute the dynamics of past devastating epidemics caused by these pathogens.

These paleomicrobiological studies have been mainly based on the classical detection of DNA sequences by using targeted PCR-sequencing [10] 16S rRNA gene PCR-sequencing [11], 16S rRNA gene PCR-NGS [12] and DNA-array-based capture and NGS [7, 13]. Later studies culminated in the reconstitution of the complete genome of ancient strains of *Y*. *pestis* in Bronze Age individuals [6] and historical plague pandemic victims [7, 14, 15], *Vibrio cholerae* [16], *Mycobacterium tuberculosis* [8], *Borrelia burgdorferi* [17] and *Treponema denticola* [18]; and host-associated viruses [19] including smallpox virus from human specimens buried for 300 years [9].

While ancient DNA decay may limit the fate of discovery of ancient microbes [20], proteins have been shown to resist alteration for hundreds of thousands of years [21, 22]. For example, no less than 126 different proteins were retrieved from the femur of a 43,000-year-old mammoth preserved in permafrost in Siberia [23]. For instance, mass spectrometry analyses of proteins resolved the question of the sheep and cattle sources of the 5,300-year-old Tyrolean Iceman’s clothes [24]. As for the discovery of microbes, this approach has been limited to the study of the dental calculus [25, 26].

We tested the hypothesis that microbes could leave identifiable protein signatures in ancient dental pulp by using ancient plague as a paradigm.

## Results

### Ancient dental pulp contains peptides

In a first step, dental pulp was collected from 16 teeth collected in 16 individuals buried in two different archaeological sites in France. These sites chosen were one negative control site without any historical, anthropological and microbiological evidence for plague (Nancy, dated 1793–1795); and one positive control site with anthropological and historical pieces of evidence of plague confirmed by previous PCR-sequencing of *Y*. *pestis* (Le Delos, dated 1720–1721) [27]. After protein extraction and purification, we observed that the concentration of proteins varied from 0.08 to 1.5 g/L. Then, mass spectrometry analysis of the 16 teeth yielded a total of 650 peptides ≥ 10 residues. The analysis of these peptides identified a total of 57 proteins in addition to trypsin used for peptidic digestion and keratins discarded as probable contaminants, whereas negative controls run in parallel yielded no peaks.

### Ancient dental pulp peptides identifying proteins of human origin

In a second step, we observed that 439 peptides were indicative of proteins of human origin, retrieved in 6/16 teeth under investigation. These peptides were indicative of a total of 30 different human proteins, comprising blood proteins (10) including immunoglobulins; connective tissue proteins (6) including collagen 1 and collagen 2; and proteins of other sources (14) like orexin and dermicin [28] (Table 1). Moreover, 14/30 proteins derived from the paleoproteomic analysis of the ancient dental pulp proteomes had been previously detected in modern-day dental pulp [29]. Keratin type 1 was assigned as a skin contaminant as keratin type 2 had been previously interpreted as a skin contamination in the modern-day dental pulp proteome [29]. In addition, five proteins detected in ancient dental pulp but not in modern-day dental pulp are deriving from blood, comprising lipocalin, immunoglobulin A and the coagulation factor.

**Table 1:** List of human proteins (except for keratins, interpreted as contaminants) identified by paleoproteomic investigations of 16 ancient dental pulp specimens collected in two archeological sites, France.

Protein	Accession number	Specimen	Peptides detected	Protein Coverage (%)
Alpha-2-HS-glycoprotein	P02765	S10	7	15,8038
Alpha-2-HS-glycoprotein	P02765	N1	4	12,5341
Amelogenin, X isoform isoform 3 precursor	NP_872621.1	S22	1	4,3902
Calmodulin-like protein 5	Q9NZT1	N1	8	75,3425
Caspase-14	P31944	N1	2	6,1983
Cerebral dopamine neurotrophic factor	Q49AH0	S22	1	21,9251
Coagulation factor IX	P00740	S10	2	4,5553
Coagulation factor X	P00742	S10	1	2,2541
Collagen alpha-1(I) chain	P02452	N1	31	24,8634
Collagen alpha-1(I) chain	P02452	S23	29	26,0929
Collagen alpha-1(I) chain	P02452	S22	9	7,7869
Collagen alpha-1(I) chain	P02452	S20	26	19,1257
Collagen alpha-1(I) chain	P02452	S10	14	13,388
Collagen alpha-2(I) chain	P08123	S20	35	17,9356
Collagen alpha-2(I) chain	P08123	S10	31	28,0381
Collagen alpha-2(I) chain	P08123	S23	31	22,7672
Collagen alpha-2(I) chain	P08123	S6	24	14,6413
Collagen alpha-2(I) chain	P08123	N1	18	13,5432
Collagen alpha-2(I) chain	P08123	S22	18	19,3997
Dermcidin	P81605	S10	3	28,1818
Dual specificity protein phosphatase 23	Q98V57	S10	1	8,6667
Galectin-7	P47929	N1	2	22,0588
Haloacid dehalogenase-like hydrolase domain-containing protein 2	NP_115500.1	S6	2	13,1274
Histone H4	P62805	S10	2	19,4175
Ig alpha-1 chain C region	P01876	S10	8	40,2266
Ig alpha-2 chain C region	P01877	S10	5	20,2941
Ig gamma-1 chain C region	P01857	N1	7	32,4242
Lipocalin-1	P31025	S10	4	35,2273
Orexin	O43612	S10	2	9,1603
Polymeric immunoglobulin receptor	P01833	S10	6	11,6492
Protein FAM104A	Q969W3	S10	2	35,4839
Protein S100-A7	P31151	N1	4	33,6634
Protein S100-A7	P31151	S23	3	21,7822
Protein S100-A8	P05109	N1	3	31,1828
Protein S100-A9	P06702	N1	5	56,1404
Protein S100-A9	P06702	S23	3	49,1228
Protein S100-A9	P06702	S10	2	24,5614
Prothrombin	P00734	S10	13	26,045
Putative lipocalin 1-like protein 1	Q5VSP4	S10	2	24,0741
RING-box protein 2	Q9UBF6	S10	2	42,4779
Serpin B3	P29508	N1	1	4,359
Serum Albumin	P02768	N1	38	54,1872
Serum Albumin	P02768	S10	26	39,9015
UV excision repair protein RAD23 homolog B	P54727	S22	1	9,5355

### Ancient dental pulp peptides identifying *Y*. *pestis* proteins.

A total of 211 peptides of bacterial origin were identified in eight ancient dental pulp specimens (S1 Table). Four peptides detected in three different individuals S16, S22 and S23 in the positive plague Delos site were found to be representative of three different proteins i.e. Blast comparisons showed that EIR43209.1, WP_002222869.1 and WP_002210283.1 exhibited 100% identity and 100% coverage with *Yersinia* spp. genome (Table 2). In particular, one peptide retrieved twice from individual S22 was found to Blast only with 100% identity and 100% coverage with *Yersinia pseudotuberculosis* and *Y*. *pestis*. No peptide similar to *Y*. *pestis* proteome was retrieved from any of the eight specimens collected in the negative control site. According to the Pearson’s chi-squared test, the probability to find peptides from *Yersinia* exclusively in the Delos site is only equal to a P value of 0.05466394 with a X^2^ indicator of 3.69230769.

**Table 2:** List of four peptides retrieved from three individuals in a documented 18^th^ century plague site, France; exhibiting 100% identity and 100% coverage (Blast on NCBI) with at least *Y*. *pestis*. * This peptide was found twice in the S22 individual.

Peptide	Specimen	Organism
(-)GIVYNPDNVADGFYYAEGGNFVQIYQYENPMFFEK(E) *	S22	*Yersinia pestis*
		*Yersinia pseudotuberculosis* complex
(K)LYDAANAALDVVDTEIAQGFPEPEWATQLREAIAEMNAPEPSEDEADWQR(F)	S16	*Yersinia pestis*
		*Buttiauxella gaviniae*
		*Enterobacter cloacae*
		*Enterobacter hormaechei*
		*Enterobacteriaceae*
		*Klebsiella oxytoca*
		*Salmonella enterica*
		*Salmonella* phage
(R)QSPEMDYFMAVFVPSFSLSLDEISLDSLD(-)	S23	*Yersinia pestis*
		*Yersinia*
		*Yersinia frederiksenii*
		*Yersinia intermedia*
		*Yersinia pseudotuberculosis*
		*Yersinia wautersii*
(R)KFNGNLNAER(I)	S23	*Yersinia pestis*
		*bacteria* symbiont BFo1 of *Frankliniella occidentalis*
		*Brenneria goodwinii*
		*Chania multitudinisentens*
		*Enterobacter ludwigii*
		*Enterobacterales*
		*Erwinia*
		*Erwinia billingiae*
		*Erwinia persicina*
		*Erwinia typogaphi*
		*Ewingella americana*
		*Pantoea ananatis*
		*Pantoea* sp.
		*Pantoea stewartii*
		*Rahnella*
		*Serratia*
		*Serratia fonticola*
		*Yersinia pseudotuberculosis*
		*Yersinia ruckeri*

## Discussion

We applied paleoproteomics to the dental pulp collected from buried individuals in order to develop a new diagnostic approach for ancient infectious diseases, using plague as an illustrative situation. Data here reported indicate that four peptides corresponding among others to *Y*. *pestis* proteins have been detected in three individuals exhumed from a documented 18^th^ century plague site in France, while no such peptide was detected in a negative control 18^th^ century site in France. In particular, one of these four peptides found twice in one individual yielded significant blast results only with *Y*. *pestis* and *Y*. *pseudotuberculosis*, which are undistinguishable when using this approach. In light of the historical, archeological and anthropological data from the archeological site of Le Delos [27] in which the presence *Y*. *pestis* was already confirmed by F1 antigen detection and suicide PCR [30, 31], we interpreted these four peptides as indicative of *Y*. *pestis* in these three individuals who died during the plague epidemic of 1720–1722.

Twenty years ago, we introduced the dental pulp as a suitable specimen on which to base the DNA detection of ancient blood-borne pathogens, chiefly *Y*. *pestis* [1, 10]. Following these pioneering works, the dental pulp has been used to recover the complete *Y*. *pestis* genome from individuals of the Bronze Age [6], medieval individuals [14] and 18^th^ century individuals [13]. Appropriate PCR-sequencing strategies also enabled us to retrieve specific DNA sequences in ancient dental pulp specimens, including the trench fever agent *Bartonella quintana* [32], the typhus agent *Rickettsia prowazekii* [33] and the typhoid fever agent *Salmonella enterica* Typhi [34].

Here, we report that the dental pulp preserves ancient peptides detectable by paleoproteomics. In this tissue, ancient peptides can be detected by highly sensitive chromatographic methods hence eliminating any amplification process and limiting potential in-laboratory contamination. Accordingly, recovered host proteins included conjunctive tissue proteins and a few blood proteins such as immunoglobulins. This expands the spectrum of retrievable inflammatory proteins previously generated by the paleoproteomic analysis of the human dental calculus [35]. Obviously, the dental calculus and the dental pulp were readily available contrary to brain tissue in which proteins had been previously analyzed in the Tyrolean Iceman [36]. The fact that immunoglobulins have been easily detected in ancient dental pulp suggests that studying ancient dental pulp proteome may give information on both the pathogen and the host inflammatory response and be used for direct and indirect serological diagnoses. This may connect with the seroepidemiology of past infections and past conditions currently diagnosed by the detection of specific immunoglobulins and other protein markers.

Not only host proteins could be recovered but also pathogen-specific ones as demonstrated in this report. As for *Y*. *pestis*, the century-long preservation of the F1 antigen in the dental pulp has been previously reported [30]. Accordingly, the pathogen and its antigens were detected in the dental pulp by using immuno-PCR [37]. Indeed, the detection of specific protein sequences is a step towards the detection of ancient pathogens. This could be of particular interest for the detection of RNA virus as the conservation of viral RNA in ancient specimens is poorly documented apart from an exceptional observation of the Ancient Northwest Territories cripavirus in 700-year-old caribou frozen feces [19].

Paleoproteomics of the dental pulp opens a new area of research in paleopathology, allowing for the diagnosis of both a blood-borne pathogen and the host inflammatory response to this pathogen.

## Materials and methods

### Ancient teeth collection

A total of 16 teeth were collected from individuals buried in two different sites in France. These teeth have been further preserved in the Regional ostéothèque, Marseille Medical School, Marseille, France.

The specimen numbers used for this study are:

For Nancy site (Usine Berger Levrault, 1794,1795): 1, 2, 3, 4, 5, 6, 7, 8

For Le Délos site (Martigues, 1720,1721): 6, 8, 10, 13, 16, 20, 22, 23

These teeth have been further preserved in the Regional ostéothèque DRAC-PACA, Marseille Medical School, North sector, Batiment A—CS80011, Bd Pierre Dramard, 13344 MARSEILLE Cedex 15, France, under the direction of Yann Ardagna and Emeline Sperandio.

No permits were required for the described study, which complied with all relevant regulations.

Eight teeth were collected from eight individuals found at the site of the Berger-Levrault factory, Nancy, used as a plague-negative control site. In 2010, rehabilitation works allowed for the discovery of a vast graveyard of the eighteenth and nineteenth centuries, part of which was the subject of an archaeological excavation operation. Historical archives indicated that this cemetery had been established *ex nihilo* in 1732; and that the excavated area dated from 1779–1842. The excavated area was located along the walls of the cemetery fence and comprised wide-trench burials for burial beds hosting several hundred individuals. Anthropological studies and archives indicated that they were French soldiers who died in a hospital setting between June 1793 and February-March 1795 [38]. Anthropological data and historical sources indicated no history of plague.

Then, eight teeth were collected from eight individuals buried in two ditches in a mass grave excavated in 1994 in the Delos site, Martigues, used as the plague-positive control site [26]. A total of 39 skeletons were exhumed (21 adults and 18 immature individuals). Historical sources indicated this was an emergency burial site dated from 1720, at a time when plague swept over Provence; and formally confirmed as a plague site by PCR-sequencing [1, 10]. Accordingly, the Delos site is one of the best characterized plague mass graves in Southern France. The dental pulp was extracted individually using new disposable instruments as previously described [1]. Extirpated dental pulps were stored no more than five days at 20°C prior to protein extraction.

### Dental pulp protein extraction

Proteins were extracted from every dental pulp specimen as previously described by Cappellini [39] with minor modifications. Briefly, the dental pulp was first powdered by sonication. Protein extraction was performed on 1.3 mg of dental pulp powder suspended in 200 μL of a 0.5 M EDTA (pH 8.01) solution incubated overnight at 4°C. After 15 min of centrifugation at maximum speed on a bench-top centrifuge at 4°C, the supernatant (referred as the EDTA fraction) was stored at -20°C. Pellets were washed twice with 200 μL of distilled water, then re-suspended in 100 μL of a 50 mM ammonium bicarbonate solution (pH 7.40) and incubated 48 h at 75°C. The specimen was then centrifuged for 15 min at maximum speed at 4°C and the supernatant (referred as bicarbonate fraction) was collected and stored at -20°C. Pellets were collected separately and re-suspended in TS buffer (urea 7M, thiourea 2M, CHAPS 4%) and incubated at 30°C for 4 h. After centrifugation for 15 min at maximum speed, the supernatant (referred as TS Fraction) was stored at -20°C. All fractions (EDTA, bicarbonate and TS) were dialyzed using Slide-ALyzer Dialysis Cassettes 2K MWCO (Pierce Biotechnology, Rockford, USA) with 2L of dialysis solution (50 mM ammonium bicarbonate Ph 7.40, urea 1M solution) for 4h. Then the dialysis solution was changed and a new dialysis was performed overnight. Dialyzed fractions were collected and quantified by Bradford Assay using Coomassie (Bradford). This mixture was reduced by 1 h-incubation at 60°C with 5 mM final concentration of dithiothreitol. The reduced cysteines were then alkylated by a 45 min-incubation in the dark at room temperature in a 15 mM iodoacetamide solution. Final pH was adjusted between 7.40 and 7.60 using concentrated sodium hydroxide. Soluble proteins were reduced and alkylated with iodoacetamide. Alkylated proteins were digested using 0.5 μg of sequencing grade trypsin (overnight incubation at 37°C). The three fractions were washed and de-salted using Detergent Removal Procedure and stored at -20°C.

### LC/MS analysis

For protein identification by LC-ESI-MS/MS, purified proteins were digested with trypsin and trypsin digests were analyzed using a nanoAcquity UPLC system connected to a Synapt G2Si Q-TOF ion mobility hybrid spectrometer. Peptides were eluted onto a trapping column (nanoAcquity UPLC 2G-V/M Trap 5μm Symmetry C18 180μm x 20mm, Waters) for concentration and desalting, at 10 μL/min of 99.9% water 0.1% formic acid and 0.1% acetonitrile 0.1% formic acid. Peptides were eluted on a C18 100 μm x 100 mm column (nanoAcquity UPLC 1.7μm BEH C18, Waters) and separated using a 100 minutes gradient (300 nl/min, 5 to 40% acetonitrile 0.1% formic acid). Data independent MS/MS monitoring (HDMSe) was performed in positive Resolution Mobility TOF mode. Capillary voltage was set to 3 kV, sampling cone to 40 V and source temperature to 90 degrees. MS range was 50–2000 m/z, Trap cell energy was 4 V, Transfer cell low energy was 5 V and high fragmentation energy was a 19–45 V ramp. Typical on-column specimen load was approximately 400 ng per specimen. Raw MS data was processed using PLGS 3.0.1 software. GFP lock mass correction was applied to all spectra. Processing thresholds were set as follow: low energy = 250 counts, elevated energy = 100 counts, intensity = 750 counts. The following workflow parameters were set for protein database searching: monoisotopic masses, 1+ minimum peptide charge, Trypsin, peptide and fragment automatic tolerances, 1 missed cleavage, carbamidomethyl C as fixed modification, deamination NQ and oxidation M as variable modification, 4% false discovery rate, 1 minimum peptide per protein, 1 minimum fragment ion matches per peptide, 3 minimum fragment ion matches per protein. NCBI and Swissprot online protein sequences were used for protein identification. All specimens MS datasets were compared to the entire Swissprot database (November 2014) and a concatenated NCBI database containing *Homo sapiens*, *Yersinia* and common environment contaminant sequences (November 2014). Keratines were excluded from the results. Proteins presenting one or more peptides were considered as identified.

### Pearson’s chi-squared test

A pearson’s chi squared test was realized between the sample of Delos and the sample of Nancy to probe the probability that the repartition of *Y*. *pestis* peptides detected had nothing to do with chance. Values used were of dd l = 1 and P<0.1for the analysis.

## Supporting information

S1 Table List of bacterial peptides identified in ancient dental pulp specimens collected in two archeological sites, France. (DOCX)
