# Supplementary material for: Paleoproteomics of the Dental Pulp: The plague paradigm
Source: PLoS One. 2017 Jul 26;12(7):e0180552. doi: 10.1371/journal.pone.0180552 (PMC5528255; doi:10.1371/journal.pone.0180552)
Supplement: Supplementary file 1 [file pone.0180552.s001.docx]

Protein	Accession number	Score	Organism	Sequence	Specimen	Peptides detected	Protein Coverage (%)	E-value
Actin-1	P02578	6,0869	*Acanthamoeba castellanii*	(K)YSVWIGGSILASLSTFQQMWISKEEYDESGPSIVHR(K)	S22	4	20,5333	4.1E-24
Actin-1	P02578	5,8902	*Acanthamoeba castellanii*	(K)LCYVALDFEQEMHTAASSSALEK(S)	S22	4	20,5333	2.8E-14
Actin-1	P02578	4,607	*Acanthamoeba castellanii*	(M)GDEVQALVIDNGSGMCK(A)	S22	4	20,5333	5.8E-8
Actin-1	P02578	4,0909	*Acanthamoeba castellanii*	(-)MGDEVQALVIDNGSGMCK(A)	S22	4	20,5333	1.7E-9
tRNA 2-thiocytidine biosynthesis protein TtcA	Q0VQ34	6,2192	*Alcanivorax borkumensis* (strain SK2 / ATCC 700651 / DSM 11573)	(R)NVVIRPLAYCREK(D)	S10	4	19,0141	1.3E-4
tRNA 2-thiocytidine biosynthesis protein TtcA	Q0VQ34	5,244	*Alcanivorax borkumensis* (strain SK2 / ATCC 700651 / DSM 11573)	(K)EMLQQWDKQQPGR(I)	S10	4	19,0141	1.7E-5
tRNA 2-thiocytidine biosynthesis protein TtcA	Q0VQ34	4,5484	*Alcanivorax borkumensis* (strain SK2 / ATCC 700651 / DSM 11573)	(K)QPGFPEHILPEYLEK(E)	S10	4	19,0141	5.9E-7
tRNA 2-thiocytidine biosynthesis protein TtcA	Q0VQ34	4,5051	*Alcanivorax borkumensis* (strain SK2 / ATCC 700651 / DSM 11573)	(R)AIADFNMISEGDK(V)	S10	4	19,0141	3.4E-4
Uncharacterized DapA-like lyase Caur_1967	A9WE23	5,9869	*Chloroflexus aurantiacus* (strain ATCC 29366 / DSM 635 / J-10-fl)	(K)MLIDSYPQLR(I)	S10	3	13,0435	8.6E-2
Uncharacterized DapA-like lyase Caur_1967	A9WE23	4,8697	*Chloroflexus aurantiacus* (strain ATCC 29366 / DSM 635 / J-10-fl)	(R)ALCDALPADAR(V)	S10	3	13,0435	1.3E-1
Uncharacterized DapA-like lyase Caur_1967	A9WE23	4,7873	*Chloroflexus aurantiacus* (strain ATCC 29366 / DSM 635 / J-10-fl)	(R)AVFDAFHQGGDVAAAQAR(L)	S10	3	13,0435	3.2E-8
Putative uncharacterized protein DDB_G0281733	Q54TI3	4,8574	*Dictyostelium discoideum*	(R)DSRDHRESRDSRDHRESRDSREYRESRDHRDHSRDSRDQYRDKDHRDSSDGRDHR(N)	S22	18	14,0794	5.4E-38
Putative uncharacterized protein DDB_G0281733	Q54TI3	4,5962	*Dictyostelium discoideum*	(R)DSRDSRDRESRDSRDHRESRDSRDHRESRDSREYRESR(D)	S22	18	14,0794	7.1E-25
Putative uncharacterized protein DDB_G0281733	Q54TI3	4,5938	*Dictyostelium discoideum*	(R)ESRDSRDHRESRDSREYRESRDHRDHSRDSRDQYRDKDHRDSSDGR(D)	S22	18	14,0794	1.4E-32
Putative uncharacterized protein DDB_G0281733	Q54TI3	4,5598	*Dictyostelium discoideum*	(R)DHRESRDSREYRESRDHRDHSRDSRDQYRDKDHRDSSDGR(D)	S22	18	14,0794	2.2E-27
Putative uncharacterized protein DDB_G0281733	Q54TI3	4,5432	*Dictyostelium discoideum*	(R)DSRDSRDSRDRESRDSRDHRESRDSRDHRESR(D)	S22	18	14,0794	1E-23
Putative uncharacterized protein DDB_G0281733	Q54TI3	4,4877	*Dictyostelium discoideum*	(R)DSRDHRESRDSREYRESRDHR(D)	S22	18	14,0794	4.9E-13
Putative uncharacterized protein DDB_G0281733	Q54TI3	4,4708	*Dictyostelium discoideum*	(R)DSSRDAKDRDSRDHRDNRDHRDSR(D)	S22	18	14,0794	1.3E-15
Putative uncharacterized protein DDB_G0281733	Q54TI3	4,3751	*Dictyostelium discoideum*	(R)DSRDHRESRDSRDHRESRDSREYRESRDHRDHSR(D)	S22	18	14,0794	8.6E-27
Putative uncharacterized protein DDB_G0281733	Q54TI3	4,3709	*Dictyostelium discoideum*	(R)DSRDHRDNRDHRDSR(D)	S22	18	14,0794	9.4E-7
Putative uncharacterized protein DDB_G0281733	Q54TI3	4,3518	*Dictyostelium discoideum*	(R)DHRDSRDYRDHRDSRDYR(D)	S22	18	14,0794	1.3E-10
Putative uncharacterized protein DDB_G0281733	Q54TI3	4,3006	*Dictyostelium discoideum*	(R)ESRDSREYRESRDHRDHSRDSR(D)	S22	18	14,0794	7.6E-14
Putative uncharacterized protein DDB_G0281733	Q54TI3	4,2576	*Dictyostelium discoideum*	(R)DSRDSRDRESRDSRDHRESRDSR(D)	S22	18	14,0794	3.2E-14
Putative uncharacterized protein DDB_G0281733	Q54TI3	4,2542	*Dictyostelium discoideum*	(R)DYRDSRDSRDSRDSRDRESRDSRDHRESRDSRDHR(E)	S22	18	14,0794	2.8E-22
Putative uncharacterized protein DDB_G0281733	Q54TI3	4,2324	*Dictyostelium discoideum*	(R)DSRDSRDSRDRESR(D)	S22	18	14,0794	8.4E-5
Putative uncharacterized protein DDB_G0281733	Q54TI3	4,2251	*Dictyostelium discoideum*	(R)DSRDYRDSRDSRDSRDSRDRESRDSR(D)	S22	18	14,0794	2.2E-17
Putative uncharacterized protein DDB_G0281733	Q54TI3	4,2069	*Dictyostelium discoideum*	(R)EYRESRDHRDHSRDSRDQYRDKDHR(D)	S22	18	14,0794	5.1E-18
Putative uncharacterized protein DDB_G0281733	Q54TI3	4,1459	*Dictyostelium discoideum*	(R)DSSRDAKDRDSRDHRDNRDHRDSRDHR(D)	S22	18	14,0794	4.6E-19
Putative uncharacterized protein DDB_G0281733	Q54TI3	4,092	*Dictyostelium discoideum*	(R)DHRDSRDYRDHRDSRDYRDSRDSRDSRDSRDRESRDSRDHRESRDSR(D)	S22	18	14,0794	1.1E-33
Putative uncharacterized protein DDB_G0281733	Q54TI3	5,4263	*Dictyostelium discoideum*	(R)RDSRDHRDSRDYRDHRDSRDYRDSRDSRDSRDSRDRESRDSRDHR(E)	S8	36	34,657	3.4E-32
Putative uncharacterized protein DDB_G0281733	Q54TI3	5,424	*Dictyostelium discoideum*	(R)DYRDHRDSRDYRDSRDSR(D)	S8	36	34,657	3.5E-10
Putative uncharacterized protein DDB_G0281733	Q54TI3	5,4161	*Dictyostelium discoideum*	(R)YYRDSSRDAKDRDSRDHRDNRDHRDSR(D)	S8	36	34,657	1.7E-19
Putative uncharacterized protein DDB_G0281733	Q54TI3	5,4058	*Dictyostelium discoideum*	(K)LQEYFNKIDYMNK(N)	S8	36	34,657	1.4E-5
Putative uncharacterized protein DDB_G0281733	Q54TI3	5,3864	*Dictyostelium discoideum*	(R)DSRDHRESRDSREYRESR(D)	S8	36	34,657	1.3E-9
Putative uncharacterized protein DDB_G0281733	Q54TI3	5,3725	*Dictyostelium discoideum*	(R)ESRDSRDHRESRDSRDHRESRDSREYRESRDHRDHSRDSRDQYR(D)	S8	36	34,657	3.1E-31
Putative uncharacterized protein DDB_G0281733	Q54TI3	5,3657	*Dictyostelium discoideum*	(R)DSRDHRESRDSRDHRESRDSREYRESRDHRDHSRDSRDQYRDKDHRDSSDGRDHR(N)	S8	36	34,657	5.4E-38
Putative uncharacterized protein DDB_G0281733	Q54TI3	5,3495	*Dictyostelium discoideum*	(M)DIFDSTITSFEDFPSER(F)	S8	36	34,657	1.7E-8
Putative uncharacterized protein DDB_G0281733	Q54TI3	5,3387	*Dictyostelium discoideum*	(R)DRESRDSRDHRESRDSRDHRESRDSREYRESRDHRDHSRDSR(D)	S8	36	34,657	5.1E-29
Putative uncharacterized protein DDB_G0281733	Q54TI3	5,3315	*Dictyostelium discoideum*	(R)DHRDSRDYRDHRDSRDYRDSRDSRDSRDSRDRESRDSRDHRESR(D)	S8	36	34,657	4.4E-31
Putative uncharacterized protein DDB_G0281733	Q54TI3	5,3298	*Dictyostelium discoideum*	(R)LDLEDSHPLDEFLELWFNFNLDNSSSSSSSSSSSSSSSSTTTTTTTTSELSSISTSLNSIR(T)	S8	36	34,657	1.3E-36
Putative uncharacterized protein DDB_G0281733	Q54TI3	5,3226	*Dictyostelium discoideum*	(R)ESRDSREYRESRDHRDHSRDSRDQYR(D)	S8	36	34,657	1.5E-18
Putative uncharacterized protein DDB_G0281733	Q54TI3	5,3037	*Dictyostelium discoideum*	(R)NSSNNNNNNNNNNNNNNNNNNNNNNNNNNNNNNNNNNNEDSGNFMNSILNYCFYEHKNHFNFIK(N)	S8	36	34,657	1E-41
Putative uncharacterized protein DDB_G0281733	Q54TI3	5,2901	*Dictyostelium discoideum*	(R)DSRDSRDRESRDSRDHRESRDSR(D)	S8	36	34,657	3.2E-14
Putative uncharacterized protein DDB_G0281733	Q54TI3	5,2373	*Dictyostelium discoideum*	(R)ESRDSRDHRESRDSREYRESRDHRDHSRDSRDQYRDKDHR(D)	S8	36	34,657	1.2E-27
Putative uncharacterized protein DDB_G0281733	Q54TI3	5,2234	*Dictyostelium discoideum*	(R)DHRDSRDYRDHRDSRDYRDSRDSRDSRDSRDRESRDSRDHRESRDSRDHRESRDSREYRESRDHR(D)	S8	36	34,657	3.5E-46
Putative uncharacterized protein DDB_G0281733	Q54TI3	5,2106	*Dictyostelium discoideum*	(R)DSRDYRDSRDSRDSRDSRDRESRDSRDHRESRDSRDHR(E)	S8	36	34,657	7.1E-25
Putative uncharacterized protein DDB_G0281733	Q54TI3	5,1914	*Dictyostelium discoideum*	(R)DSRDHRDSRDYRDHRDSRDYRDSRDSRDSRDSRDRESRDSRDHRESRDSRDHRESRDSREYRESRDHRDHSRDSR(D)	S8	36	34,657	1.1E-54
Putative uncharacterized protein DDB_G0281733	Q54TI3	5,1428	*Dictyostelium discoideum*	(R)DYRDSRDSRDSRDRESRDSRDHRESR(D)	S8	36	34,657	7.9E-21
Putative uncharacterized protein DDB_G0281733	Q54TI3	5,1332	*Dictyostelium discoideum*	(R)DSRDSRDRESRDSRDHRESRDSRDHRESRDSREYRESRDHRDHSRDSRDQYRDK(D)	S8	36	34,657	2.2E-36
Putative uncharacterized protein DDB_G0281733	Q54TI3	5,1139	*Dictyostelium discoideum*	(R)RDSRDHRDSRDYRDHRDSRDYRDSRDSRDSRDSRDRESRDSRDHRESRDSRDHRESRDSR(E)	S8	36	34,657	2E-41
Putative uncharacterized protein DDB_G0281733	Q54TI3	5,0917	*Dictyostelium discoideum*	(R)DYRDHRDSRDYRDSRDSRDSRDSRDRESRDSRDHRESRDSRDHRESRDSREYRESRDHR(D)	S8	36	34,657	7.5E-41
Putative uncharacterized protein DDB_G0281733	Q54TI3	5,0391	*Dictyostelium discoideum*	(R)DHRDSRDYRDSRDSRDSRDSRDRESRDSRDHRESRDSRDHRESRDSREYRESRDHRDHSRDSR(D)	S8	36	34,657	3.7E-44
Putative uncharacterized protein DDB_G0281733	Q54TI3	5,036	*Dictyostelium discoideum*	(R)DSRDRESRDSRDHRESRDSRDHRESRDSREYRESRDHRDHSRDSRDQYRDKDHRDSSDGRDHR(N)	S8	36	34,657	1.3E-44
Putative uncharacterized protein DDB_G0281733	Q54TI3	5,0303	*Dictyostelium discoideum*	(R)DSRDSRDSRDRESRDSRDHRESRDSRDHRESR(D)	S8	36	34,657	1.1E-21
Putative uncharacterized protein DDB_G0281733	Q54TI3	5,0284	*Dictyostelium discoideum*	(R)ESRDSRDHRESRDSRDHRESRDSREYRESRDHRDHSRDSRDQYRDKDHRDSSDGRDHR(N)	S8	36	34,657	1.9E-40
Putative uncharacterized protein DDB_G0281733	Q54TI3	5,026	*Dictyostelium discoideum*	(R)DSRDSRDRESRDSRDHRESRDSRDHRESRDSREYRESRDHRDHSR(D)	S8	36	34,657	3.2E-34
Putative uncharacterized protein DDB_G0281733	Q54TI3	5,0187	*Dictyostelium discoideum*	(R)DSRDRESRDSRDHRESRDSRDHRESRDSREYRESRDHRDHSRDSRDQYRDKDHR(D)	S8	36	34,657	2.8E-39
Putative uncharacterized protein DDB_G0281733	Q54TI3	4,9988	*Dictyostelium discoideum*	(R)DSRDHRDSRDYRDHRDSRDYR(D)	S8	36	34,657	1.3E-13
Putative uncharacterized protein DDB_G0281733	Q54TI3	4,9901	*Dictyostelium discoideum*	(R)DHRDSRDYRDHRDSRDYRDSRDSRDSRDSRDRESRDSRDHRESRDSRDHRESRDSREYR(E)	S8	36	34,657	7.5E-41
Putative uncharacterized protein DDB_G0281733	Q54TI3	4,9838	*Dictyostelium discoideum*	(R)DHRESRDSRDHRESRDSR(E)	S8	36	34,657	1.9E-9
Putative uncharacterized protein DDB_G0281733	Q54TI3	4,9833	*Dictyostelium discoideum*	(K)LDYEVYDSFK(S)	S8	36	34,657	1.3E-1
Putative uncharacterized protein DDB_G0281733	Q54TI3	4,9649	*Dictyostelium discoideum*	(R)DYRDSRDSRDSRDSRDRESRDSRDHRESRDSRDHRESRDSREYR(E)	S8	36	34,657	1.2E-30
Putative uncharacterized protein DDB_G0281733	Q54TI3	4,9202	*Dictyostelium discoideum*	(R)DHRDSRDYRDHRDSRDYRDSRDSRDSRDSRDRESRDSRDHR(E)	S8	36	34,657	1.9E-25
Putative uncharacterized protein DDB_G0281733	Q54TI3	4,949	*Dictyostelium discoideum*	(R)DYRDSRDSRDSRDSRDRESRDSRDHRESRDSRDHRESRDSR(E)	S8	36	34,657	1.8E-27
Putative uncharacterized protein DDB_G0281733	Q54TI3	4,9662	*Dictyostelium discoideum*	(R)DHRDSRDYRDSRDSRDSRDSRDRESRDSRDHRESRDSRDHRESRDSREYRESRDHR(D)	S8	36	34,657	4.1E-38
HssA/B-like protein 26	Q86H91	5,0518	*Dictyostelium discoideum*	(K)IGNIKSSSSSSIGSGK(S)	S8	3	79,5699	3.1E-5
HssA/B-like protein 26	Q86H91	5,7638	*Dictyostelium discoideum*	(R)IACGECGGSATGLLGNIANSGTGMVSQIPVTVMLDVNANANLNPSMSAVPAGNSCGCN(-)	S8	3	79,5699	6E-41
HssA/B-like protein 26	Q86H91	5,8151	*Dictyostelium discoideum*	(R)IACGECGGSATGLLGNIANSGTGMVSQIPVTVMLDVNANANLNPSMSAVPAGNSCGCN(-)	S8	3	79,5699	6E-41
HssA/B-like protein 33	P15648	4,537	*Dictyostelium discoideum*	(M)TLFSSISSMSSSMTSSR(S)	S10	1	17,3469	1.6E-7
Putative uncharacterized protein DDB_G0281733	Q54TI3	4,9464	*Dictyostelium discoideum*	(R)ESRDHRDHSRDSRDQYRDKDHRDSSDGR(D)	S10	19	14,9218	3.6E-20
Putative uncharacterized protein DDB_G0281733	Q54TI3	4,8597	*Dictyostelium discoideum*	(R)RDSRDHRDSRDYRDHR(D)	S10	19	14,9218	3.9E-8
Putative uncharacterized protein DDB_G0281733	Q54TI3	4,8382	*Dictyostelium discoideum*	(R)ESRDHRDHSR(D)	S10	19	14,9218	2.4E-1
Putative uncharacterized protein DDB_G0281733	Q54TI3	4,6847	*Dictyostelium discoideum*	(R)DHSRDSRDQYRDK(D)	S10	19	14,9218	1.4E-4
Putative uncharacterized protein DDB_G0281733	Q54TI3	4,6787	*Dictyostelium discoideum*	(R)DSSRDAKDRDSR(D)	S10	19	14,9218	1.7E-2
Putative uncharacterized protein DDB_G0281733	Q54TI3	4,6326	*Dictyostelium discoideum*	(R)DSRDHRESRDSREYRESRDHRDHSRDSRDQYRDK(D)	S10	19	14,9218	4.4E-27
Putative uncharacterized protein DDB_G0281733	Q54TI3	4,6112	*Dictyostelium discoideum*	(R)DSRDHRESRDSRDHRESRDSR(E)	S10	19	14,9218	1.9E-12
Putative uncharacterized protein DDB_G0281733	Q54TI3	4,5916	*Dictyostelium discoideum*	(R)ESRDSRDHRESRDSREYRESRDHRDHSRDSRDQYRDK(D)	S10	19	14,9218	1.2E-24
Putative uncharacterized protein DDB_G0281733	Q54TI3	4,5808	*Dictyostelium discoideum*	(R)ESRDSRDHRESRDSREYRESRDHRDHSRDSRDQYR(D)	S10	19	14,9218	5.4E-23
Putative uncharacterized protein DDB_G0281733	Q54TI3	4,5579	*Dictyostelium discoideum*	(R)DSRDHRESRDSRDHR(E)	S10	19	14,9218	1.8E-6
Putative uncharacterized protein DDB_G0281733	Q54TI3	4,5378	*Dictyostelium discoideum*	(R)DSRDSRDRESRDSRDHR(E)	S10	19	14,9218	3.2E-8
Putative uncharacterized protein DDB_G0281733	Q54TI3	4,5184	*Dictyostelium discoideum*	(R)DHRDSRDYRDHRDSRDYRDSRDSRDSR(D)	S10	19	14,9218	1.2E-19
Putative uncharacterized protein DDB_G0281733	Q54TI3	4,4938	*Dictyostelium discoideum*	(R)DHRDHSRDSRDQYR(D)	S10	19	14,9218	6,E-06
Putative uncharacterized protein DDB_G0281733	Q54TI3	4,4842	*Dictyostelium discoideum*	(R)DNRDHRDSRDHR(D)	S10	19	14,9218	8.9E-4
Putative uncharacterized protein DDB_G0281733	Q54TI3	4,4193	*Dictyostelium discoideum*	(R)EYRESRDHRDHSRDSRDQYRDKDHRDSSDGRDHR(N)	S10	19	14,9218	3.1E-27
Putative uncharacterized protein DDB_G0281733	Q54TI3	4,4121	*Dictyostelium discoideum*	(R)YYRDSSRDAKDRDSRDHRDNR(D)	S10	19	14,9218	4.9E-13
Putative uncharacterized protein DDB_G0281733	Q54TI3	4,3775	*Dictyostelium discoideum*	(R)DHRDHSRDSRDQYRDKDHRDSSDGR(D)	S10	19	14,9218	3.8E-17
Putative uncharacterized protein DDB_G0281733	Q54TI3	4,3757	*Dictyostelium discoideum*	(R)DSRDYRDSRDSR(D)	S10	19	14,9218	3.3E-3
Putative uncharacterized protein DDB_G0281733	Q54TI3	4,3589	*Dictyostelium discoideum*	(R)DSRDSRDSRDRESRDSRDHRESRDSR(D)	S10	19	14,9218	3,E-20
Putative uncharacterized protein DDB_G0271982	Q86AH4	5,4492	*Dictyostelium discoideum*	(K)EKEKEKEKEKEKEKEIEKEREKERE
